# Identification of key genes in the pathogenesis of preeclampsia via bioinformatic analysis and experimental verification

**DOI:** 10.3389/fendo.2023.1190012

**Published:** 2023-07-28

**Authors:** Yongqi Gao, Zhongji Wu, Simin Liu, Yiwen Chen, Guojun Zhao, Hui-Ping Lin

**Affiliations:** ^1^ Department of Basic Medical Research, The Sixth Affiliated Hospital of Guangzhou Medical University, Qingyuan People’s Hospital, Key Laboratory of Cardiovascular Diseases, School of Basic Medical Sciences, Guangzhou Medical University, Guangzhou, China; ^2^ The Sixth Affiliated Hospital of Guangzhou Medical University, Qingyuan City People’s Hospital, Qingyuan, Guangdong, China

**Keywords:** bioinformatics, preeclampsia, differentially expressed genes, biomarkers, therapeutic targets

## Abstract

**Background:**

Preeclampsia (PE) is the primary cause of perinatal maternal-fetal mortality and morbidity. The exact molecular mechanisms of PE pathogenesis are largely unknown. This study aims to identify the hub genes in PE and explore their potential molecular regulatory network.

**Methods:**

We downloaded the GSE148241, GSE190971, GSE74341, and GSE114691 datasets for the placenta and performed a differential expression analysis to identify hub genes. We performed Gene Ontology (GO), Kyoto Encyclopedia of Genes and Genomes (KEGG), Disease Ontology (DO), Gene Set Enrichment Analysis (GSEA), and Protein–Protein Interaction (PPI) Analysis to determine functional roles and regulatory networks of differentially expressed genes (DEGs). We then verified the DEGs at transcriptional and translational levels by analyzing the GSE44711 and GSE177049 datasets and our clinical samples, respectively.

**Results:**

We identified 60 DEGs in the discovery phase, consisting of 7 downregulated genes and 53 upregulated genes. We then identified seven hub genes using Cytoscape software. In the verification phase, 4 and 3 of the seven genes exhibited the same variation patterns at the transcriptional level in the GSE44711 and GSE177049 datasets, respectively. Validation of our clinical samples showed that CADM3 has the best discriminative performance for predicting PE

**Conclusion:**

These findings may enhance the understanding of PE and provide new insight into identifying potential therapeutic targets for PE.

## Introduction

Preeclampsia (PE) is a pregnancy-related disease that occurs after the 20th gestational week, characterized by clinical symptoms such as hypertension, proteinuria, poor placental vascularization, abnormal maternal cardiovascular adaptations, and fetal growth restriction. PE is the leading cause of maternal and fetal mortality and morbidity worldwide, affecting up to 10% of pregnancies (1, 2). The underlying mechanism and preventive treatment for PE are still under investigation.

With the rapid development of next-generation sequencing, the exploration of diagnostic and therapeutic biomarkers for PE has made significant progress. Bioinformatics analysis of these big data provides new leads for identifying reliable and functional differentially expressed genes and transcripts. Reanalyzing these big data from various medical sources might provide novel insights from other perspectives and evidence for mapping molecular pathogenesis networks of disease. In recent years, several independent studies on EOPE (early-onset pre-eclampsia) using RNA sequencing technology and bioinformatics analysis have identified dysregulated pathways, including the G-protein coupled receptor (GPCR) signaling pathway, endocytosis pathway, the focal adhesion pathway, as well as abnormal expression of multiple miRNAs and mRNAs (3–5). These findings provide important insights into the pathophysiology of EOPE. However, due to small sample sizes, the improper grouping of clinical subtypes, and insufficient analyzing the transcriptome data, studies failed to identify distinct molecular markers in PE.

In the present study, we analyzed four publicly available microarray datasets of early-onset eclampsia retrieved from Gene Expression Omnibus (GEO), an array- and sequence-based database repository submitted by the research community. We used computational approaches to identify differentially expressed genes (DEGs) associated with PE and conducted enrichment analysis and protein-protein interaction (PPI) networks. We then another two validation cohorts to validate dysregulated expressions from GEO datasets. Further, we then used RT-PCR to test the mRNA expression levels of the identified genes using PE and control samples from clinical, expecting to provide potential biomolecules for early detection of PE, subsequent clinical treatment, and more fully understand the pathogenesis mechanism of PE.

## Materials and methods

### Expression profile dataset selection

We searched for the gene expression profile data of early-onset PE (EOPE) in the GEO database and screened the identified datasets according to the inclusion and exclusion criteria. The inclusion criteria were as follows (1): including five or more pairs of samples from the EOPE group and normal group (2), the Dataset type is expression profiling by array or RNA profiling by array (3),the sample type is the human placenta. The exclusion criteria were as follows: no complete gene expression profile data was provided. Finally, six datasets related to EOPE in placenta tissue transcriptome data were retrieved through the GEO database. The information on these datasets was displayed in **Table 1**. GSE74341, GSE114691, GSE148241, and GSE190971 datasets were used for bioinformatic analysis. GSE177049 and GSE44711 were used for validation of the bioinformatic analysis.

**Table 1:** Data resource.

ID	Status(N:P)	Organism	Tissue	Platform	Analysis or Validation
GSE148241	32:9	Homo sapiens	Placenta	GPL16791	Analysis
GSE190971	6:7	Homo sapiens	Placenta	GPL11154	Analysis
GSE74341	10:7	Homo sapiens	Placenta	GPL16699	Analysis
GSE114691	21:20	Homo sapiens	Placenta	GPL11154	Analysis
GSE44711	8:8	Homo sapiens	Placenta	GPL10558	Validation
GSE177049	6:6	Homo sapiens	Placenta	GPL24676	Validation

### Data processing and differentially expressed genes identification

Raw microarray data for six datasets were downloaded from the GEO database. We normalized the data using the R package limma (6) and converted it to log_2_ values for further analysis. We used the R package limma to obtain DEGs between the EOPE and control groups from GSE74341, GSE114691, GSE148241, and GSE190971 datasets. Genes with p < 0.05 and |log_2_ fold change (FC)| ≥ 1 were considered DEGs in the respective databases. The overlapping DEGs among the four datasets were considered the final DEGs. The volcano maps and Venn diagrams were generated through the Sangerbox 3.0 (http://sangerbox.com/) (7).

### Function and pathway analysis of DEGs

Gene ontology analysis (GO) is an effective method for annotating genes and identifying characteristic biological attributes, including biological processes (BP), molecular functions (MF), and cellular components (CC) (8). The Kyoto Encyclopedia of Genes and Genomes (KEGG) database offers a thorough collection of data on protein interaction networks and bio-interpretation of genomic sequences (9). Genes are annotated in Disease Ontology (DO) with links to human diseases (10). In our study, GO, KEGG, and DO enrichment analysis of DEGs were completed by the ‘clusterProfiler’ package in R software (11). For all the cases, we recorded all the enriched terms with p-value < 0.05.

### Gene set enrichment analyse of datasets

Gene Set Enrichment Analysis (GSEA) is a gene set-based functional pathway enrichment analysis method that calculates the enrichment fraction of gene sets in each functional pathway (12). GSEA was performed using GSEABase, clusterProfiler, and org.Hs.eg.db packages. Differences at p-value < 0.05 were defined as the cutoff criteria.

### PPI network analysis

PPI network analysis of DEGs was performed using the online STRING website (https://string-db.org/) (13). Then, use Cytoscape software (10) to visualize the PPI network. The screening condition for constructing the PPI network was a combined score > 0.3.

### Identifying the key module and hub genes

Molecular Complex Detection (MCODE) is a plugin for Cytoscape to build important functional modules in PPI networks (14). Parameters were set as Node Score Cutoff = 0.2, Degree Cutoff = 2, K-Core = 4, and Max. Depth = 100. Moreover, three algorithms, namely, Density of Maximum Neighborhood Component (DMNC), Maximum Neighborhood Component (MNC), and Clustering Coefficient, were used in the cytoHubba plugin (15) to determine the top 12 hub genes. The genes that were present in all three algorithm results were considered the final hub genes. In addition, a sub-network of hub genes was generated from the PPI network.

### Verification of hub gene expression by validation datasets

The validation datasets were processed in the same way as the analysis datasets. The mRNA level of the hub genes was validated in the GSE177049 and GSE44711 datasets.

### Validation of clinical specimens

Quantitative reverse transcriptase-PCR (RT-PCR) was used for the quantitative expression of hub genes. Placentas were collected from 18 clinical samples, including 9 healthy controls and 9 with EOPE. The cycle threshold (CT) data were determined, and the mean CT was determined from triplicate PCRs. Relative gene expression was calculated with the equation 2^–ΔCT^.

### Construction of miRNAs-hub genes network and TFs-hub genes network

Target miRNAs of the hub genes were predicted with the miRTarBase (16), Starbase (17), and Targetscan (18) databases. To improve the prediction accuracy, We chose predicted miRNAs for each hub gene, which were predicted by at least two databases. Predicted miRNAs that regulate multiple hub genes were considered critical miRNAs. We submitted the hub genes to ChIP-X Enrichment Analysis 3 (ChEA3) platform for TF prediction (19). Hub genes-associated TFs were ranked by mean rank score. Finally, we selected the TFs which score ≤ 50 as key predicted TFs. The miRNAs-hub genes network and TFs-hub genes network were visualized using Cytoscape.

### Statistical analysis

We used R software and GraphPad Prism to conduct statistical analyses. For RNA-seq datasets, we utilized the R package limma to identify DEGs. To determine key modules and hub genes in the protein-protein interaction network, we employed two commonly used Cytoscape plugins, MCODE and cytoHubba software. In the verification analysis, the normality of continuous variables was assessed using the Shapiro-Wilk test, and the homogeneity of variances was tested using Levene’s test. For normally distributed data, two-tailed t-tests were performed. For non-normally distributed data, the Mann-Whitney U test was used. p<0.05 was considered to be statistically significant.

## Results

### Identification of DEGs in PE

**Figure 1:**
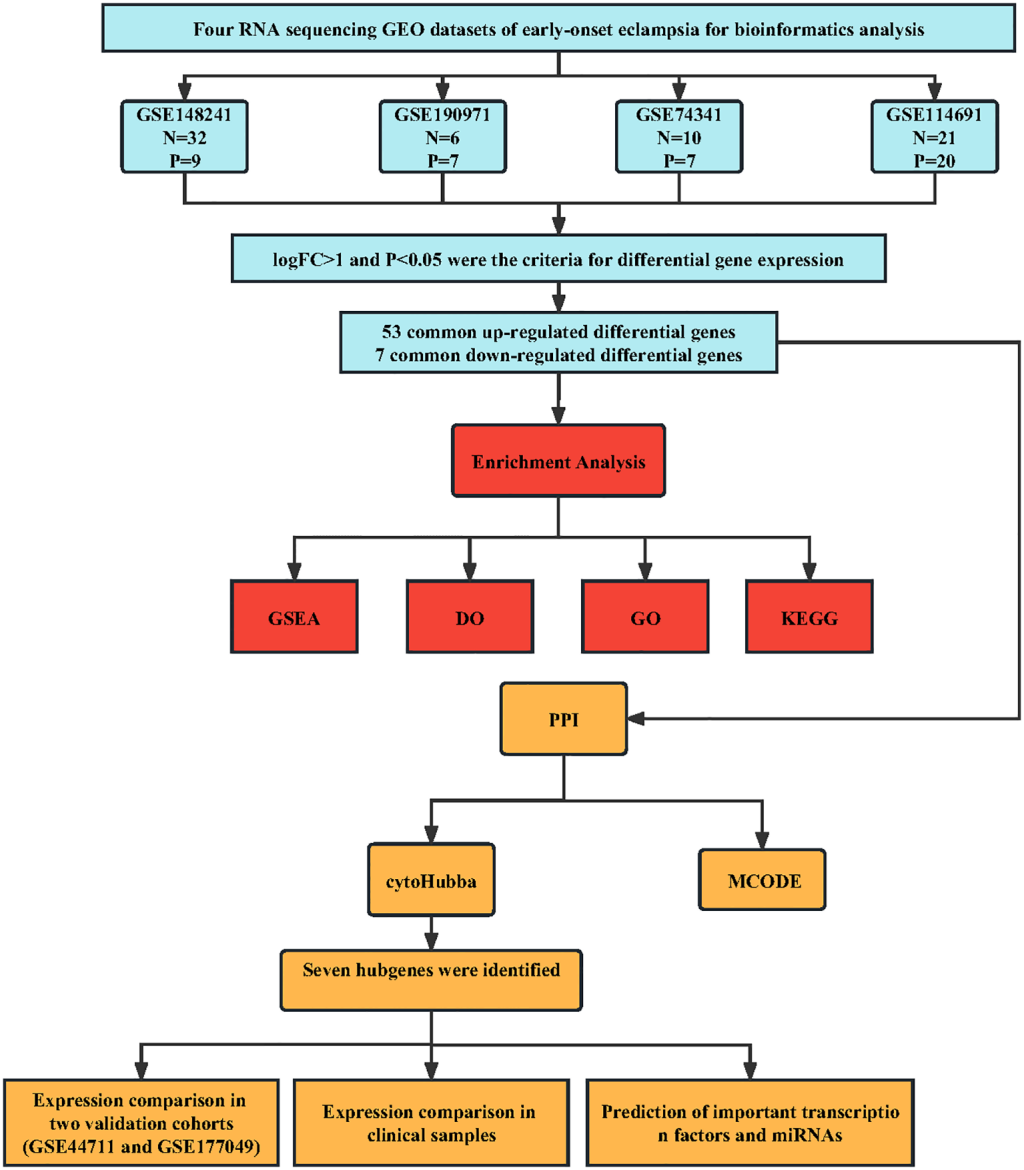
The flowchart of the analysis process.

**Figure 2:**
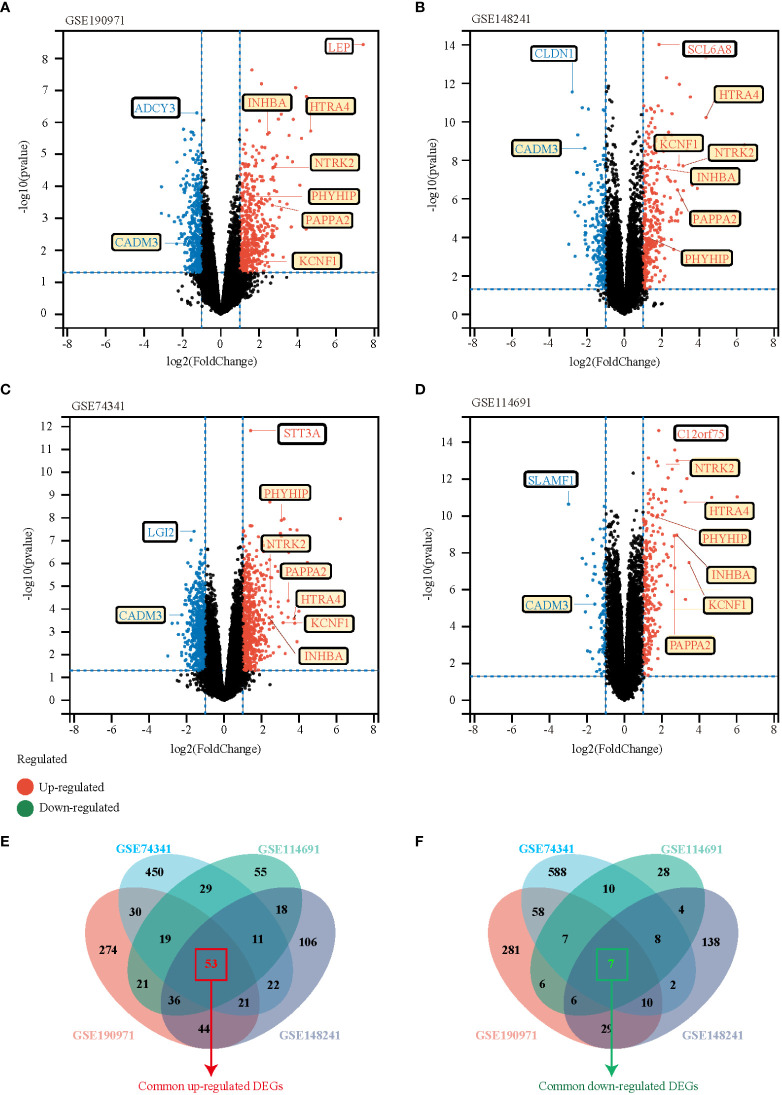
Identification of DEGs between the normal group and EOPE group in the analysis datasets. **(A-D)** The volcano plots of DEGs in GSE190971, GSE148241, GSE74341, and GSE114691, respectively. Red dots indicate genes with high expression levels, blue dots indicate genes with low expression levels, and black dots indicate genes with no differential expression based on the criteria of p-value < 0.05 and |log 2 fold change (FC)| ≥ 1, respectively. **(E, F)** Venn diagrams showed the overlaps of numbers of upregulated **(E)** and down-regulated **(F)** DEGs between the four analysis datasets.

We performed a systematic review of GEO datasets to identify the DEGs between normal pregnant women and women with PE. A schematic representation of the screening strategy to identify such compounds is shown in **Figure 1**. We selected four acceptable datasets, GSE148241, GSE190971, GSE74341, and GSE114691, which comprised at least 5 pairs of normal or PE placenta expression profiling by RNA array (**Table 1**). Based on the criteria of |log_2_ FC|> 1 and P < 0.05 of the data preprocessing specified in the Materials and Methods section, we identified and visualized the DEGs in related datasets by volcano mapping and heat mapping in the respective databases (**Figures 2A–D**; **Supplementary Figure 1**). We draw a Venn diagram showing the DEGs from the 4 datasets. As shown in **Figures 2E, F**, we obtained a total of 60 DEGs, of which 7 downregulated genes and 53 upregulated genes (**Supplementary Table 1**).

### GO, KEGG, and DO enrichment results of DEGs

**Figure 3:**
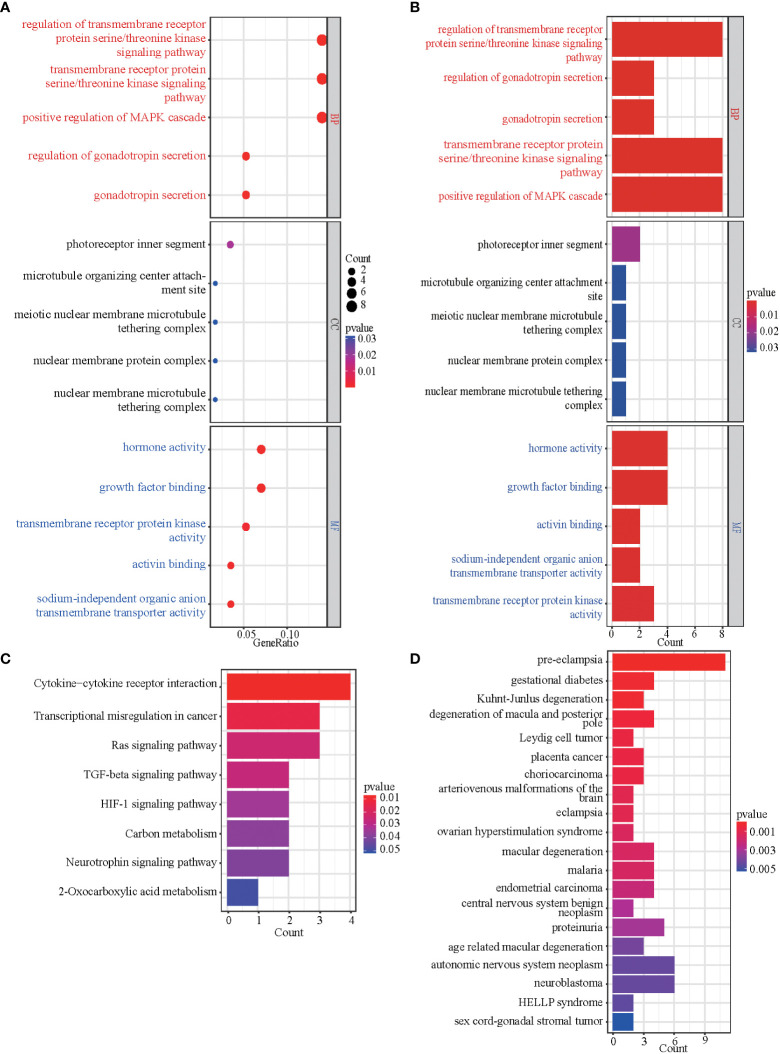
GO, KEGG, and DO enrichment results of DEGs. **(A, B)** The bubble plot and bar graph showed the top 5 significant items in the BP, CC, and MF fractions based on the P-values in the GO analysis. **(C)** The bar graph showed the result of the KEGG enrichment analysis. **(D)** The bar graph showed the result of DO enrichment.

To better understand the function of the overlapping genes, we subjected the 60 overlapping genes to GO, KEGG, DO, and GSEA analyses. According to the results of GO analysis results, the changes in biological processes (BP) of DEGs were significantly enriched in the ‘regulation of transmembrane receptor protein serine/threonine kinase signaling pathway,’ ‘transmembrane receptor protein serine/threonine kinase signaling pathway,’ ‘positive regulation of MAPK cascade,’ ‘regulation of gonadotropin secretion,’ and ‘gonadotropin secretion’; the changes in cell component (CC) of DEGs were enriched in ‘photoreceptor inner segment,’ ‘microtubule organizing center attachment site,’ ‘meiotic nuclear membrane microtubule tethering complex,’ ‘nuclear membrane protein complex,’ and ‘nuclear membrane microtubule tethering complex’; and the changes in molecular function (MF) were enriched in ‘hormone activity,’ ‘growth factor binding,’ ‘transmembrane receptor protein kinase activity,’ ‘activin binding,’ and ‘sodium-independent organic anion transmembrane transporter activity.’ (**Figures 3A, B**). KEGG pathway analysis revealed that the pathways enriched by dysregulated DEGs include ‘cytokine-cytokine receptor interaction,’ ‘transcriptional misregulation in cancer,’ ‘Ras signaling pathway,’ ‘TGF-beta signaling pathway,’ and ‘HIF-1 signaling pathway’ (**Figure 3C**). DO analysis revealed that the diseases enriched by dysregulated DEGs include ‘preeclampsia,’ ‘gestational diabetes,’ ‘Kuhnt-Junlus degeneration of macula and posterior pole,’ ‘Leydig cell tumor,’ and ‘placenta cancer’ (**Figure 3D**). The top five GO terms and all KEGG and DO pathways are displayed visually (**Figure 3**). Moreover, we utilized GSEA to quantify the potential functional pathway (**Table 2**).

**Table 2:** Summarize GSEA results of analysis datasets.

ID	Description	Regulation	Analysis datasets
hsa00513	Various types of N-glycan biosynthesis	Up	GSE74341,GSE114691,GSE190971
hsa04371	Apelin signaling pathway	Down	GSE74341;GSE114691;GSE148241;GSE190971
hsa04613	Neutrophil extracellular trap formation	Down	GSE74341;GSE114691;GSE148241;GSE190971
hsa04921	Oxytocin signaling pathway	Down	GSE74341;GSE114691;GSE148241;GSE190971
hsa00532	Glycosaminoglycan biosynthesis - chondroitin sulfate/dermatan sulfate	Down	GSE74341;GSE114691;GSE148241
hsa04022	cGMP-PKG signaling pathway	Down	GSE74341;GSE114691;GSE190971
hsa04260	Cardiac muscle contraction	Down	GSE74341;GSE114691;GSE148241
hsa04261	Adrenergic signaling in cardiomyocytes	Down	GSE74341;GSE114691;GSE148241
hsa04270	Vascular smooth muscle contraction	Down	GSE74341;GSE114691;GSE190971
hsa04310	Wnt signaling pathway	Down	GSE74341;GSE114691;GSE148241
hsa04512	ECM-receptor interaction	Down	GSE74341;GSE114691;GSE148241
hsa04540	Gap junction	Down	GSE74341;GSE114691;GSE148241
hsa04610	Complement and coagulation cascades	Down	GSE74341;GSE114691;GSE148241
hsa04611	Platelet activation	Down	GSE74341;GSE114691;GSE190971
hsa04621	NOD-like receptor signaling pathway	Down	GSE74341;GSE114691;GSE190971
hsa04670	Leukocyte transendothelial migration	Down	GSE74341;GSE148241;GSE190971
hsa04713	Circadian entrainment	Down	GSE74341;GSE114691;GSE148241
hsa04725	Cholinergic synapse	Down	GSE74341;GSE114691;GSE148241
hsa04933	AGE-RAGE signaling pathway in diabetic complications	Down	GSE74341;GSE114691;GSE148241
hsa05032	Morphine addiction	Down	GSE74341;GSE114691;GSE148241
hsa05143	African trypanosomiasis	Down	GSE74341;GSE148241;GSE190971
hsa05144	Malaria	Down	GSE74341;GSE114691;GSE148241
hsa05412	Arrhythmogenic right ventricular cardiomyopathy	Down	GSE74341;GSE114691;GSE148241
hsa05414	Dilated cardiomyopathy	Down	GSE74341;GSE114691;GSE148241

### Protein-protein interaction network analysis

**Figure 4:**
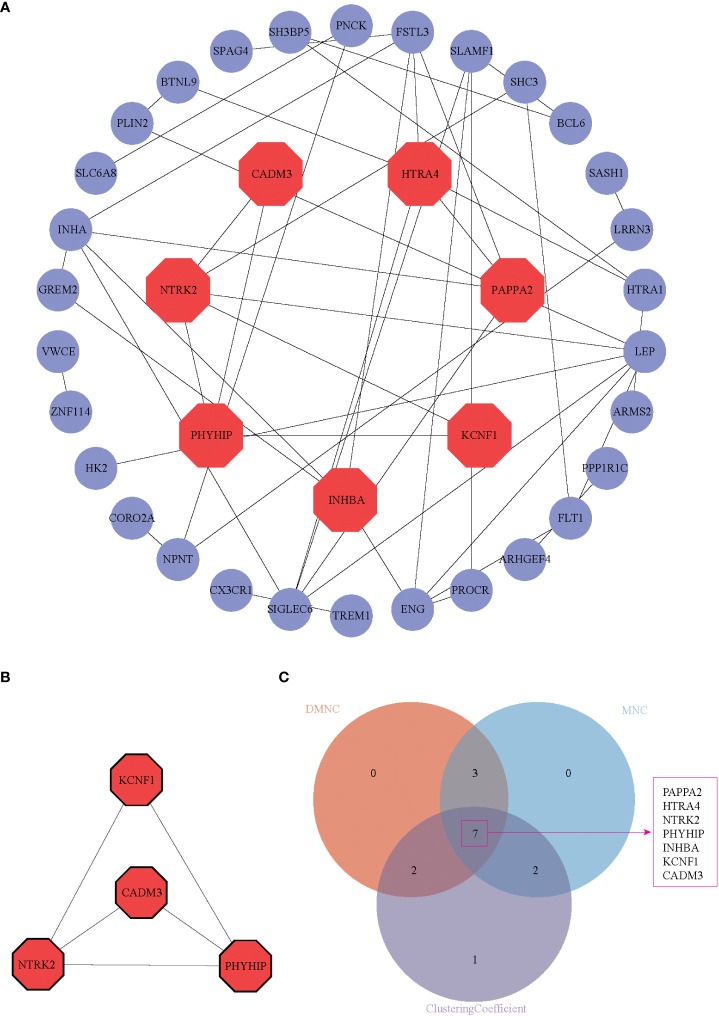
PPI network, hub genes identification, and function module identification. **(A)** PPI network displayed 37 DEGs in the PPI network after hiding disconnected nodes in the network, **(B)** One key module was identified based on MCODE analysis. **(C)** Seven hub genes were present in three algorithms simultaneously (DMNC, MNC, and Clustering Coefficient). These seven genes were shown in red octagon in **(A)**.

We uploaded DEGs to the STRING online database to form the protein-protein interaction network. We used the Cytoscape software to generate a PPI network. With a PPI score > 0.3, we built a PPI network with 37 DEGs after hiding disconnected nodes (**Figure 4A**). Next, we identified one key module based on MCODE analysis (MCODE score = 3.333), including four key DEGs, CADM3, KCNF1, NTRK2, and PHYHIP (**Figure 4B**). We also identified seven hub genes (**Figure 4C**), which included the 4 DEGs from MCODE analysis and the other three genes (PAPPA2, HTRA4, and INHBA) in three algorithms simultaneously (DMNC, MNC, and Clustering Coefficient).

### Dataset and clinical validation of 7 hub genes expression

**Figure 5:**
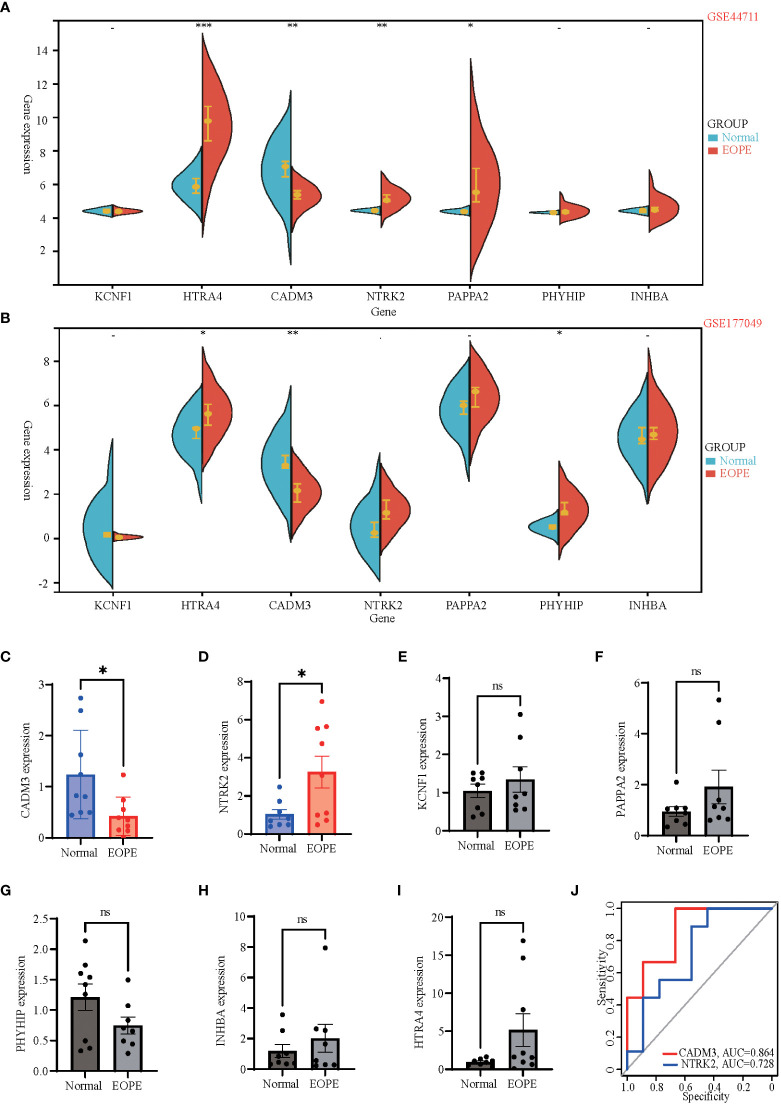
Dataset and clinical validation of 7 hub genes. **(A, B)** Split violin plots exhibiting expression intensity and expression levels of 7 hub genes in GSE44711 **(A)** and GSE177049 **(B)**. Blue represents normal placenta tissue, while red represents the placenta tissue of EOPE. **(C-I)** Validation of differential mRNA expression of hub genes between EOPE and normal placentas in local clinical samples. **(J)** The ROC curves showed the diagnostic value of CADM3 and NTRK2 in EOPE, based on local clinical samples, with AUC values shown below the curves. Data shown mean ± SEM. *n = 9* *p < 0.05, **p < 0.01, ***p < 0.001; ns: p > 0.05.

To validate the critical genes identified by PPI analysis, we examined the seven potential key genes in the GEO database GSE44711 and GSE177049 (**Table 1**). **Figure 5A** showed the violin plots of differentially expressed genes in GSE44711, where genes HTRA4, NTRK2, and PAPPA2 were significantly upregulated, while CADM3 was downregulated (**Figure 5A**). We further revealed the relationship between the expression of these genes using the database GSE177049. The expression of HTRA4 and PHYHIP genes were significantly upregulated, while gene CADM3 was significantly downregulated (**Figure 5B**). Next, we collected placental tissues from normal pregnant women and women with PE, and via RT-PCR, we investigated the relative gene expression levels of the seven genes. We observed that CADM3 showed decreased expression while NTRK2 showed increased expression levels in the PE group relative to their expression levels in the normal group (**Figures 5C–I**). To further evaluate the relationship between these seven hub genes and PE, we used the GSE190971 dataset, which has patient blood pressure information, to validate the correlation between the hub gene expression and the highest blood pressure. As shown in **Supplementary Figure 2**, CADM3 was negatively correlated to the patient’s blood pressure, while all the other six hub genes positively correlated to the patient’s blood pressure. We also plotted ROC curves and AUC values to evaluate hub genes’ sensitivity and specificity for EOPE diagnosis. The results indicated both CADM3 were highly accurate in EOPE diagnosis, and the AUC was 0.864 (**Figure 5J**).

### miRNAs-hub genes and TFs-hub genes network

**Figure 6:**
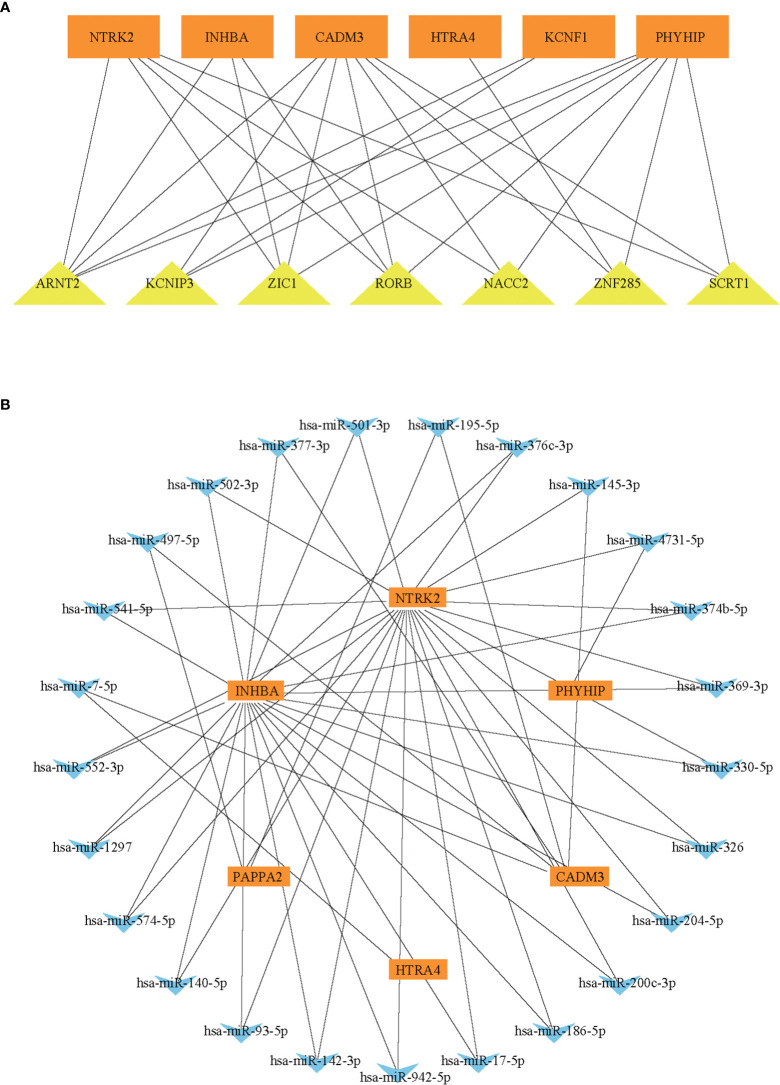
The predicted miRNAs-hub genes regulatory network and predicted TFs-hub genes regulatory network. **(A)** The TFs-hub genes regulatory network. **(B)** The miRNA-hub genes regulatory network.

To further explore the regulatory function of the hub genes, we predicted target miRNAs from three online database and TFs from one online database. We submitted the hub genes to ChIP-X Enrichment Analysis 3 (ChEA3) platform for TF prediction (19). Hub genes-associated TFs were ranked by mean rank score. Finally, we selected the TFs which score ≤ 50 as key predicted TFs. We identified seven TFs, ARNT2, KCNIP3, ZIC1, RORB, NACC2, ZNF285, and SCRT1 (**Figure 6A**). We predicted target miRNAs of the hub genes using the miRTarBase (16), Starbase (17), and Targetscan (18) databases. To improve the accuracy of the prediction, We chose predicted at least two databases miRNAs for each hub gene. Predicted critical miRNAs regulate multiple hub genes (**Figure 6B**).

## Discussion

Preeclampsia is divided into early-onset preeclampsia and late-onset preeclampsia, based on gestational age at diagnosis or delivery. Early-onset preeclampsia, less common than late-onset preeclampsia but with more severe clinical onset features, threatens maternal and fetal health worldwide, especially in developing countries (20–22). The pathogenesis of EOPE is unclear, and an ideal early clinical biomarker for the prediction of EOPE are lacking. Several studies used bioinformatics analysis techniques to uncover potential biomarkers and cellular signaling pathways in preeclampsia (23, 24); however, in most of these studies, preeclampsia was not classified as early-onset eclampsia and late-onset eclampsia. In contrast to previous studies that only analyzed a single dataset (25, 26), this study combined four GEO datasets related to EOPE for bioinformatics analyses to explore potential signaling pathways and biomarkers of EOPE. In addition, we used two other GEO datasets associated with EOPE and local clinical samples to validate the biomarkers.

Disease Ontology (DO) analysis for the 60 DEGs revealed that PE had the highest enrichment score, indicating that the DEGs obtained in this study were strongly associated with preeclampsia. We further used KEGG and GO to validate the DEGs. Our analysis demonstrated that multiple enriched signaling pathways are related to the pathogenesis of PE. A previous study revealed that serine/threonine kinases are involved in the placental inflammatory response (27) and are the most common signaling pathway when analyzing the fetal genes with severe PE (28). Our data supported the hypothesis that serine/threonine kinase may affect the EOPE patient and their offspring. Both previous studies and our results demonstrated that gonadotropin regulation and secretion (29, 30), Cytokine-cytokine receptor interaction (31), the activation of the Ras signaling pathway and MAPK signaling pathway (32), TGF-β signaling pathway (33), HIF-1 signaling pathway (34, 35), and 2-Oxocarboxylic acid metabolism (36, 37) are critical pathways for PE development.

We also performed a GSEA analysis on each database in this study to obtain more potential pathways. Unlike KEGG and GO, GSEA was performed on the complete gene expression profile and obtained signaling pathways that may be up- or down-regulated when a positive phenotype occurs. The present study combined GSEA results from four databases and hypothesized a potential link between the Apelin signaling pathway and eclampsia. Although no previous eclampsia study mentioned the Apelin signaling pathway, studies reported in amniotic cells (38) and primordial trophoblast cells (39) that silencing the Apelin signaling pathway promoted the release of inflammatory factors, including IL-1β, IL-6, and IL-8, which provide critical roles in multiple aspects of PE development (40–42). In the present study, we speculate that down-regulation of the Apelin signaling pathway induced the release of these inflammatory cytokines from placental tissue and promoted the development of EOPE.

The present study identified 7 DEGs (PAPPA2, HTRA4, NTRK2, PHYHIP, INHBA, KCNF1, and CADM3) as hub genes that play essential roles in EOPE. The validated datasets and our confirmation experiments using the local clinical placenta samples demonstrated that the mRNA expression of PAPPA2, HTRA4, NTRK2, PHYHIP, and CADM3 genes differ between EOPE and healthy maternal placenta. Previous studies reported that PAPPA2 and HTRA4 are biomarkers of EOPE (43–45), while no such studies regarding PHYHIP, NTRK2, and CADM3 for EOPE, indicate these three DEGs might be new biomarkers of the disease. Our data showed for the first time that the mRNA expression of NTRK2 is higher in the EOPE placenta, while NTRK2(TrkB) protein is significantly higher in female depression patients than in the healthy control group (46). There’s a link between PE and postpartum depression (PPD), recent studies have shown that 20.5% of women with PE or eclampsia suffer from postpartum depression (47), women who had PE had nearly 3-fold increased odds of PPD compared to normal women, and the risk of PPD increased with the aggravation of PE (48). Taken together, we speculate that overexpression of NTRK2 in EOPE patients may account for the vulnerability of PPD in EOPE patients, but this needs to be verified in follow-up studies. Our data also revealed that CADM3 mRNA was down-regulated in EOPE patients. CADM3 codes for the cell adhesion molecule 3 protein, also called nectin-like molecule 1 (necl-1). CADM3 is a tumor suppressor gene, while the functions of many tumor suppressor genes on eclampsia have different results (49–51), the role of CADM3 on early eclampsia remains unclear. CADM3 may be a complementary and innovative point in the study of the tumor suppressor genes in the pathogenesis of EOPE. In the present study, we verified that NTRK2 and CADM3 were biomarkers of EOPE, revealed that NTRK2 and CADM3 had good diagnostic efficacy, especially CADM3, whose area under ROC curve (AUC) was up to 86.4%, indicating CADM3 is a good diagnostic marker for EOPE. Moreover, we also found a strong negative correlation between the expression of CADM3 mRNA and the maximum blood pressure of pregnant women, suggesting that CADM3 may also be related to the severity of EOPE.

To further investigate the regulatory mechanism of hub genes in EOPE, we predicted miRNAs and TFs regulating hub genes based on the prediction databases. We noticed that ARNT2 gene, a TF that can simultaneously target five hub genes. Previous studies showed that ARNT2 mRNA expression is increased in the placenta of PE patients (52), it involved in the PE developmental classic pathway (HIF-1 signaling pathway) (53); in addition, the up-regulation of BCL6-ARNT2 pathway increased the sensitive of trophoblast to ischemia and hypoxia and increased the expression of FLT1 (52), which is a major driver of elevated blood pressure in the end-stage PE pathway (54). We also predicted multiple miRNAs that targets new biomarker CADM3, including miR-195-5p, whose expression was increased in PE placenta (55). In endothelial cells incubated with PE plasma, miR-195-5p expression is elevated, which leads to decreased VEGFA expression and decreased angiogenesis (56).

It must be emphasized that we relied on previously published datasets in this study. The suggested pathogenic mechanisms of miR-195-5p and CADM3 related to EOPE needs further validation in cell and/or animal model.

## Data availability statement

The datasets presented in this study can be found in online repositories. The names of the repository/repositories and accession number(s) can be found in the article/**Supplementary Material**.

## Ethics statement

The studies involving human participants were reviewed and approved by Guangzhou First People’s Hospital. The patients/participants provided their written informed consent to participate in this study.

## Author contributions

HL conceived and supervised the study. YG wrote the original draft of the manuscript. GZ and HL revised the manuscript. YG, ZW, SL, and YC performed the experiments and analyzed the data. All authors contributed to the article and approved the submitted version.
