# Supplementary material for: Identification of key genes in the pathogenesis of preeclampsia via bioinformatic analysis and experimental verification
Source: Front Endocrinol (Lausanne). 2023 Jul 28;14:1190012. doi: 10.3389/fendo.2023.1190012 (PMC10420078; doi:10.3389/fendo.2023.1190012)
Supplement: Supplementary file 1 [file DataSheet_1.pdf]

Title: Identification of key genes in the pathogenesis of preeclampsia via bioinformatic analysis and experimental verification

Authors: Yongqi Gao[1], Zhongji Wu[1], Simin Liu[1], Yiwen Chen[1], Guojun Zhao[2*], Huiping Lin[1*].

Affiliations:

[1]Department of Basic Medical Research, The Sixth Affiliated Hospital of Guangzhou Medical University, Qingyuan People's Hospital, Key Laboratory of Cardiovascular Diseases, School of Basic Medical Sciences, Guangzhou Medical University, Guangzhou 511518, P.R. China.

[2]The Sixth Affiliated Hospital of Guangzhou Medical University, Qingyuan City People's Hospital, Qingyuan, Guangdong, China.

* Correspondance: zhaoguojun@gzhmu.edu.cn (G.J. Z.) ; 2021390022@gzhmu.edu.cn (H.P. L.).

Supplementary Figures and Table

Supplementary Table 1, Venn DEGs

Gene	Up or down regulation	Gene	Up or down regulation
LEP	Up regulation	ARNT2	Up regulation
TREM1	Up regulation	NPNT	Up regulation
HTRA4	Up regulation	SASH1	Up regulation
PAPPA2	Up regulation	PADI1	Up regulation
SPAG4	Up regulation	HTRA1	Up regulation
KCNF1	Up regulation	SYDE1	Up regulation
PHYHIP	Up regulation	SLCO2A1	Up regulation
QPCT	Up regulation	CORO2A	Up regulation
PPP1R1C	Up regulation	PROCR	Up regulation
GREM2	Up regulation	GUCA2A	Up regulation
SH3PXD2A	Up regulation	SLCO4A1	Up regulation
FSTL3	Up regulation	CST6	Up regulation
MYO7B	Up regulation	SLC6A8	Up regulation
SIGLEC6	Up regulation	VWCE	Up regulation
BTNL9	Up regulation	BCL6	Up regulation
HK2	Up regulation	SHC3	Up regulation
PLIN2	Up regulation	TPBG	Up regulation
ARHGEF4	Up regulation	PNCK	Up regulation
INHA	Up regulation	TBC1D26	Up regulation
NTRK2	Up regulation	NDRG1	Up regulation
NEK11	Up regulation	CP	Up regulation
KRT15	Up regulation	WDR86	Up regulation
ENG	Up regulation	SFXN3	Up regulation
NPFFR2	Up regulation	LRRN3	Down regulation
ZNF114	Up regulation	RALYL	Down regulation
INHBA	Up regulation	SLAMF1	Down regulation
ARMS2	Up regulation	CX3CR1	Down regulation
SH3BP5	Up regulation	MXRA5	Down regulation
FLT1	Up regulation	KCNK17	Down regulation
GPT2	Up regulation	CADM3	Down regulation

Supplementary Figure 1,

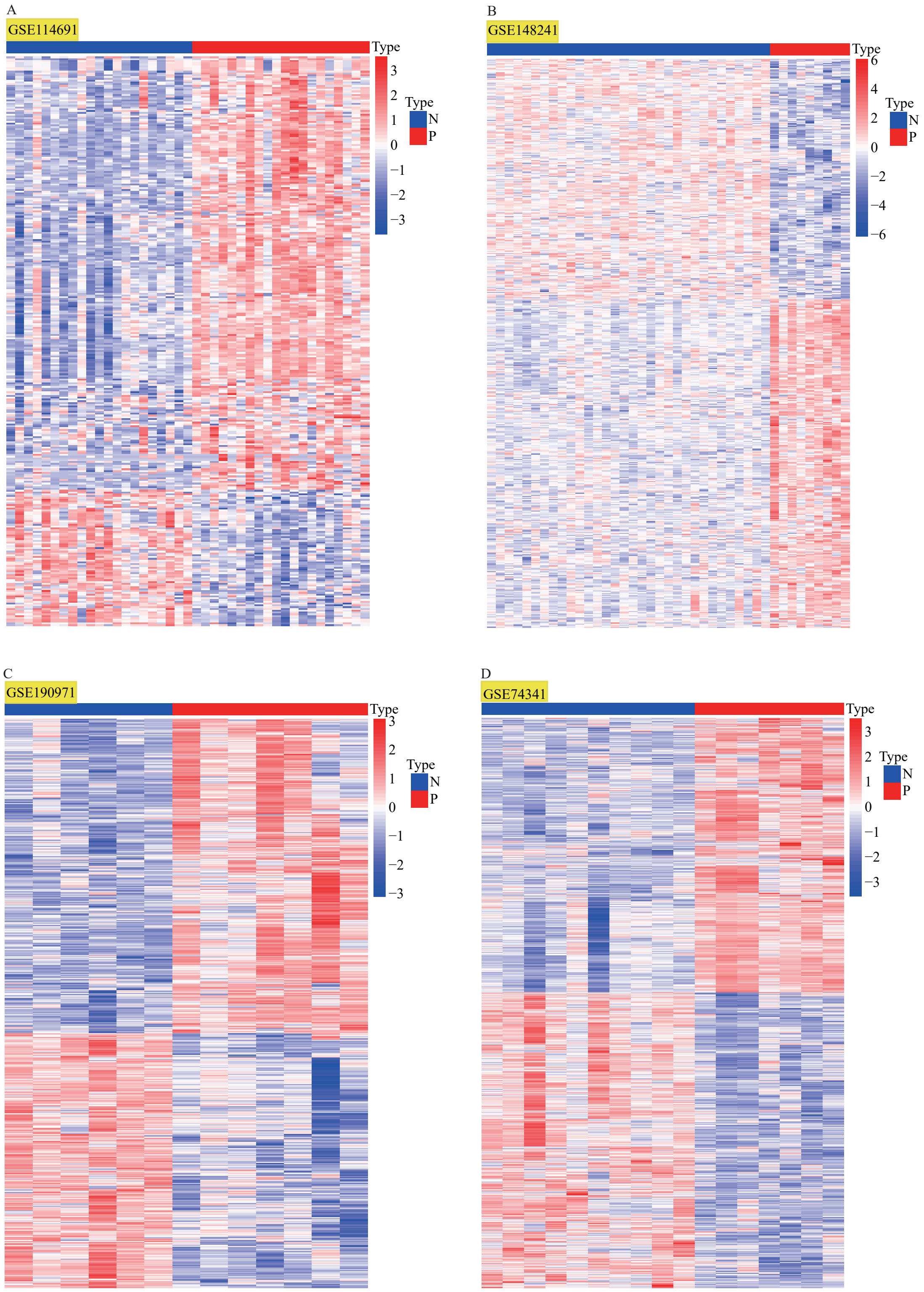

Supplementary Figure 1 Heatmaps for all DEGs between EOPE and healthy samples from GSE114691(A), GSE148241(B), GSE190971(C), and GSE74341(D). Red: Up-regulation; Blue: Down-regulation.

Supplementary Figure 2

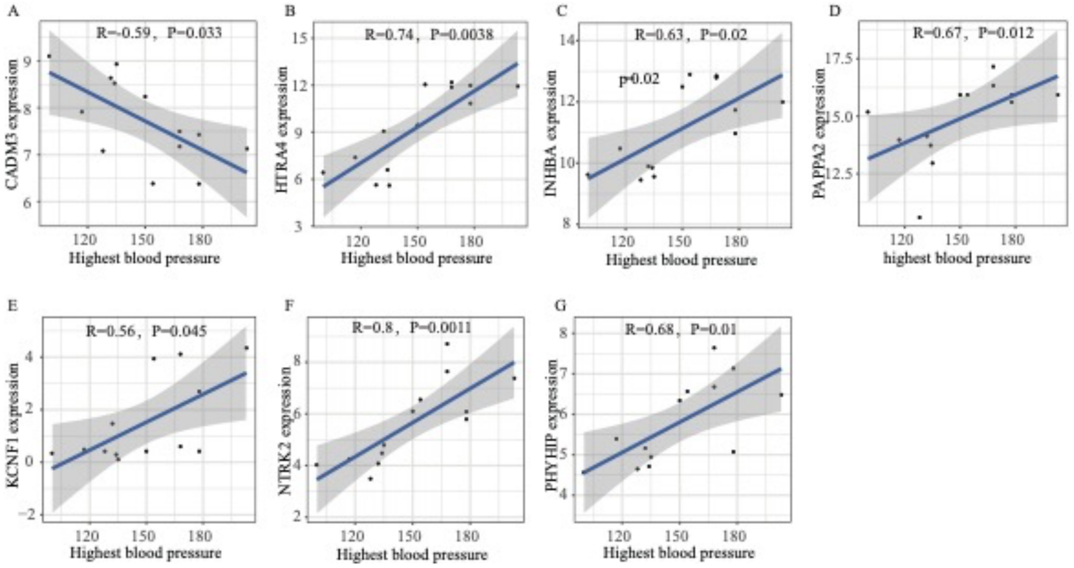

Supplementary Figure 2. Correlation scatter plots showed the correlation between the hub gene expression and the highest blood pressure in GSE190971 dataset.
