# Supplementary material for: Identification of key genes in the pathogenesis of preeclampsia via bioinformatic analysis and experimental verification
Source: Front Endocrinol (Lausanne). 2023 Jul 28;14:1190012. doi: 10.3389/fendo.2023.1190012 (PMC10420078; doi:10.3389/fendo.2023.1190012)
Supplement: Supplementary file 2 [file DataSheet_2.pdf]

```
#Note: Gene annotation of the gene matrix before performing
differential gene expression analysis
#Load the relevant R package
library(ggplot2)
library(limma)
library(pheatmap)
library(ggsci)
library(dplyr)
lapply(c('clusterProfiler','enrichplot','patchwork'), function(x)
{library(x, character.only = T)})

GSE="GSE74341"     #This code uses GSE74341 as an example
C="C"              #Name of normal control group
P="P"              #Name of disease experimental group
Ccol="blue"        #Heat map comment bar normal group colour
Pcol="red"         #Heat map annotation bar disease group colours
setwd("Fill in the location where the gene matrix is stored here")
rt=read.table("GSE74341_matrix.txt",header = T,sep="\t")
rt=as.matrix(rt)
rownames(rt)=rt[,1]
exp=rt[,2:ncol(rt)]
dimnames=list(rownames(exp),colnames(exp))
rt=matrix(as.numeric(as.matrix(exp)),nrow=nrow(exp),dimnames=dimname
s)
rt=avereps(rt)

#Create group files beforehand
sample=read.table("sample.txt",sep="\t",header=F,check.names=F,row.n
ames = 1)
rt=rt[,rownames(sample)]
#Check the number of people in groups C and P
Cgroup=sum(sample[,1]==C)
Pgroup=sum(sample[,1]==P)

#Determine if the original data is converted to log2, if max(rt) is
greater than 30 then it needs to be converted to log2
max(rt)
if(max(rt)>50) rt=log2(rt+1)

#Data normalisation using normalizeBetweenArrays
rt1=normalizeBetweenArrays(as.matrix(rt))
data=rt1

#Differential expression analysis using the limma package
conData=data[,as.vector(colnames(data)[sample[,1]==C])]
treatData=data[,as.vector(colnames(data)[sample[,1]==P])]
rt=cbind(conData,treatData)
conNum=ncol(conData)
treatNum=ncol(treatData)
Type=c(rep("con",conNum),rep("treat",treatNum))
design <- model.matrix(~0+factor(Type))
colnames(design) <- c("con","treat")
fit <- lmFit(rt,design)
cont.matrix<-makeContrasts(treat-con,levels=design)
```

```
fit2 <- contrasts.fit(fit, cont.matrix)
fit2 <- eBayes(fit2)
Diff=topTable(fit2,adjust='fdr',number=length(rownames(data)))

#Preservation of differential genes that meet the criteria
DIFFOUT=rbind(id=colnames(Diff),Diff)
diffSig=Diff[with(Diff, (abs(logFC)>1 & P.Value < 0.05 )), ]
diffSigOut=rbind(id=colnames(diffSig),diffSig)
write.table(diffSigOut,file=paste0("2.","differential
genes.xls"),sep="\t",quote=F,col.names=F)
#Up to this point, we have obtained DEGs for the GSE74341 data.
#Thank you.
```
